# Oxygen torus and its coincidence with EMIC wave in the deep inner magnetosphere: Van Allen Probe B and Arase observations

**DOI:** 10.1186/s40623-020-01235-w

**Published:** 2020-08-03

**Authors:** M. Nosé, A. Matsuoka, A. Kumamoto, Y. Kasahara, M. Teramoto, S. Kurita, J. Goldstein, L. M. Kistler, S. Singh, A. Gololobov, K. Shiokawa, S. Imajo, S. Oimatsu, K. Yamamoto, Y. Obana, M. Shoji, F. Tsuchiya, I. Shinohara, Y. Miyoshi, W. S. Kurth, C. A. Kletzing, C. W. Smith, R. J. MacDowall, H. Spence, G. D. Reeves

**Affiliations:** 1 Institute for Space-Earth Environmental Research, Nagoya University, Nagoya, Japan; 2 Graduate School of Science, Kyoto University, Kyoto, Japan; 3 Graduate School of Science, Tohoku University, Sendai, Japan; 4 Advanced Research Center for Space Science and Technology, Kanazawa University, Kanazawa, Japan; 5 Department of Space Systems Engineering, Kyushu Institute of Technology, Kitakyusyu, Japan; 6 Research Institute for Sustainable Humanosphere, Kyoto University, Uji, Japan; 7 Space Science and Engineering Division, Southwest Research Institute, San Antonio, TX USA; 8 University of Texas at San Antonio, San Antonio, TX USA; 9 Institute for the Study of Earth, Oceans, and Space, University of New Hampshire, Durham, NH USA; 10 Indian Institute of Geomagnetism, Navi Mumbai, India; 11 North-Eastern Federal University, Yakutsk, Russia; 12 Graduate School of Science, The University of Tokyo, Tokyo, Japan; 13 Faculty of Engineering, Osaka Electro-Communication University, Neyagawa, Japan; 14 Institute of Space and Astronautical Science, Japan Aerospace Exploration Agency, Sagamihara, Japan; 15 Department of Physics and Astronomy, University of Iowa, Iowa City, IA USA; 16 Solar System Exploration Division, Goddard Space Flight Center, Greenbelt, MD USA; 17 Space Sciences and Applications Group, Los Alamos National Laboratory, Los Alamos, NM USA

**Keywords:** Oxygen torus, EMIC wave, ULF wave, Ion composition, Inner magnetosphere

## Abstract

We investigate the longitudinal structure of the oxygen torus in the inner magnetosphere for a specific event found on 12 September 2017, using simultaneous observations from the Van Allen Probe B and Arase satellites. It is found that Probe B observed a clear enhancement in the average plasma mass (*M*) up to 3–4 amu at *L* = 3.3–3.6 and magnetic local time (MLT) = 9.0 h. In the afternoon sector at MLT ~ 16.0 h, both Probe B and Arase found no clear enhancements in *M*. This result suggests that the oxygen torus does not extend over all MLT but is skewed toward the dawn. Since a similar result has been reported for another event of the oxygen torus in a previous study, a crescent-shaped torus or a pinched torus centered around dawn may be a general feature of the O^+^ density enhancement in the inner magnetosphere. We newly find that an electromagnetic ion cyclotron (EMIC) wave in the H^+^ band appeared coincidently with the oxygen torus. From the lower cutoff frequency of the EMIC wave, the ion composition of the oxygen torus is estimated to be 80.6% H^+^, 3.4% He^+^, and 16.0% O^+^. According to the linearized dispersion relation for EMIC waves, both He^+^ and O^+^ ions inhibit EMIC wave growth and the stabilizing effect is stronger for He^+^ than O^+^. Therefore, when the H^+^ fraction or *M* is constant, the denser O^+^ ions are naturally accompanied by the more tenuous He^+^ ions, resulting in a weaker stabilizing effect (i.e., larger growth rate). From the Probe B observations, we find that the growth rate becomes larger in the oxygen torus than in the adjacent regions in the plasma trough and the plasmasphere.
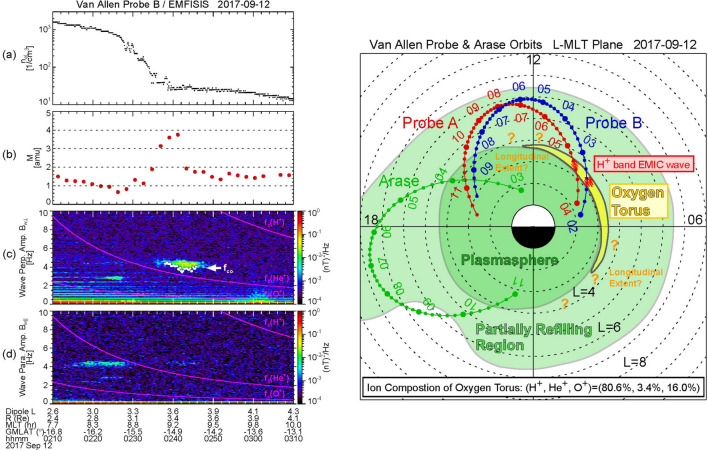

## Introduction

The inner magnetospheric O^+^ density enhancement (known as the dense oxygen torus) was first discovered by Chappell (1982) employing the retarding ion mass spectrometer (RIMS) instrument onboard the Dynamic Explorer (DE) 1 satellite. Its radial extent has been reported by many studies indicating that the oxygen torus is formed around *L* = 3–5 near the plasmapause during a storm recovery phase (Chappell, 1982; Comfort et al. 1988; Horwitz et al. 1984, 1986, 1990; Roberts et al. 1987). However, its longitudinal extent is yet uncertain. Despite its name, there has been no observational evidence that the oxygen torus extends over all longitudes. Using ~ 2-year (from late 1981 until early 1984) data from the DE-1/RIMS instrument, Roberts et al. (1987) conducted a statistical study about the magnetic local time (MLT) distribution of the oxygen torus and found that its occurrence frequency has peaks in the late evening and dawn regions (20–24 MLT and 05–09 MLT). Here, it should be noted that this statistical result may include a dependence on the geomagnetic storm phase and the F10.7 index. Thus, a case study is also needed to confirm the longitudinal extent of the oxygen torus. A recent study employing the Arase and Van Allen Probes A satellites at different MLT examined the longitudinal extent for the event of 24 April 2017 (Nosé et al. 2018). In this event, Arase flying in the morning sector detected an enhancement of the average plasma mass up to ~ 3.5 amu around *L *= 4.9–5.2 and MLT = 5.0 h, while Probe A flying in the afternoon sector observed no clear enhancements in the average plasma mass. Therefore, Nosé et al. (2018) concluded that the oxygen torus does not extend over all MLT and it is rather skewed toward the dawn sector, being described more precisely as a crescent-shaped torus or a pinched torus.

The dense O^+^ ions in the inner magnetosphere will change the dispersion relation of the electromagnetic ion cyclotron (EMIC) waves and may play an important role in excitation of such waves. Yu et al. (2015) found that the preferred region for the occurrence of O^+^ band EMIC waves in the predawn to noon sector, which is generally consistent with that of the oxygen torus reported by Roberts et al. (1987). They supposed that the oxygen torus is a vital factor responsible for the generation of EMIC waves in the O^+^ band. From a series of linear instability analyses and hybrid simulations, Min et al. (2015) found that an increase in the cold O^+^ concentration enhances the growth rate of the O^+^ band EMIC waves. On the other hand, Yuan et al. (2019) reported that richer heavy ion compositions in low-density regions (~ a few hundreds of cm^−3^) suppress growth of EMIC waves. Further studies are needed to reveal a possible relation between the oxygen torus and EMIC wave excitation.

O^+^ ions can be accelerated to higher than a few tens of keV during magnetic field dipolarization in the inner magnetosphere (Fu et al. 2002; Nosé et al. 2010, 2014, 2016; Ohtani et al. 2007). Since ions having energy of > a few tens of keV carry a major portion of the energy density of the ring current (e.g., Williams 1981), the accelerated O^+^ ions can predominantly contribute to the ring current population. Thus, the dense oxygen torus is considered a “reservoir” of low energy O^+^ ions that are accelerated during storm time substorms to form the O^+^-rich ring current (e.g., Hamilton et al. 1988; Daglis 1997). It is also possible that dense O^+^ ions have an influence on dayside magnetic reconnection (e.g., Garcia et al. 2010; Wiltberger 2015, Wiltberger et al. 2010), when they drift to the dayside magnetopause. Existence of the dense O^+^ ions increases the average plasma mass to more than 1 amu, resulting in reduction of the Alfvén wave velocity. This will affect various phenomena occurring in the magnetosphere (for example, standing waves along the geomagnetic field, magnetospheric/plasmaspheric cavity resonance, and Kelvin–Helmholtz instability), because the Alfvén wave velocity is a fundamental parameter of plasma. We, therefore, think that the study of the oxygen torus, in particular, revealing its spatial distribution and ion composition, is important for precise understanding of magnetospheric physics.

In the present study, we focus on simultaneous observations of the magnetic field and plasma waves made by the Van Allen Probe B and Arase satellites (Mauk et al. 2013; Miyoshi et al. 2018a) on 12 September 2017. In this event, the orbital configuration of the satellites is opposite to that for the 24 April 2017 event examined by Nosé et al. (2018); that is, Probe B was in the morning and Arase was in the afternoon. We will investigate if the longitudinal extent of the oxygen torus for this event is similar to that for the 24 April 2017 event. It is of great interest to study whether EMIC waves are excited within the oxygen torus in this event.

The paper is organized as follows. “Simultaneous observations by Van Allen Probe B and Arase on 12 September 2017” section demonstrates simultaneous measurements of the Van Allen Probe B and Arase satellites during the recovery phase of a magnetic storm that occurred on 12 September 2017. The average plasma mass was enhanced up to 3–4 amu in the morning sector according to the Probe B observations, while it was almost constant at 1–1.5 amu in the afternoon. There was a coincidence between the H^+^ band EMIC wave occurrence and the enhancement of the average plasma mass in the morning. In “Discussion” section we discuss the longitudinal structure of the oxygen torus, the ion composition of the oxygen torus, and the effect of the oxygen torus on EMIC wave excitation. Ion flux data in the low energy range (1 eV–50 keV) will be used to ensure the existence of the oxygen torus. Observations by Probe A are also introduced and discussed. “Conclusions” section summarizes findings of the present study.

## Simultaneous observations by Van Allen Probe B and Arase on 12 September 2017

### Satellite orbits and geomagnetic conditions

**Fig. 1:**
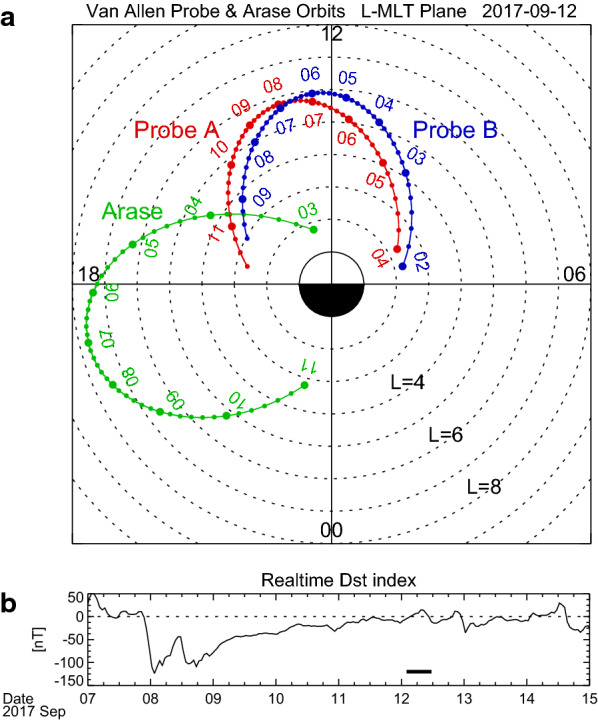
**a** Orbits of Van Allen Probe A for 04:00–11:30 UT (red curve), Van Allen Probe B for 02:00–09:30 UT (blue curve), and Arase for 03:00–11:00 UT (green curve) on 12 September 2017 in the *L*-MLT plane. **b** The real-time Dst index for 7–14 September 2017. A horizontal bar represents the time interval of 02:00–11:30 UT on 12 September 2017 which covers the orbital segments of Probe A, Probe B, and Arase shown in **a**

Figure 1a shows the orbits of Van Allen Probe A for 04:00–11:30 UT (red curve), Van Allen Probe B for 02:00–09:30 UT (blue curve), and Arase for 03:00–11:00 UT (green curve) on 12 September 2017. Probes A and B were flying from morning to afternoon through noon where they had their apogee. Arase started from afternoon, traversed dusk with an apogee around MLT = 19 h, and arrived at premidnight. In this section, data from Probe B and Arase will be examined in detail. Later, in “Observation by Van Allen Probe A” section, observations by Probe A will be briefly introduced to justify results obtained from the Probe B data.

Figure 1b presents the real-time Dst index for 7–14 September 2017 (World Data Center for Geomagnetism, Kyoto et al. 2015). A magnetic storm occurred on 7 September and reached at Dst_min_ = –124 nT at 01 UT on 8 September. A horizontal bar indicates the time interval of the orbital segments shown in Fig. 1a (i.e., 02:00–11:30 UT on 12 September). We found that this event occurred during the recovery phase of the magnetic storm in the same way as the 24 April 2017 event.

### Signatures of standing Alfvén waves and upper hybrid resonance waves

**Fig. 2:**
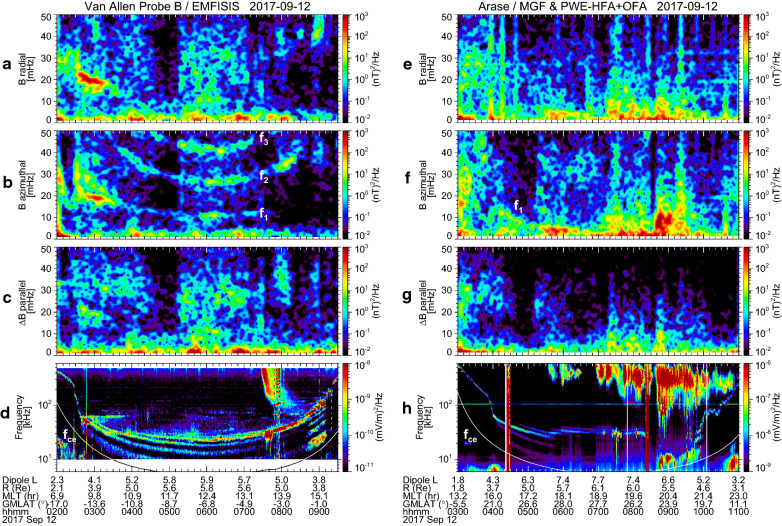
**a**–**d** Van Allen Probe B observations during 02:00–09:30 UT on 12 September 2017. The top three panels display the dynamic power spectra of the magnetic field variations in the radial, azimuthal, and compressional components measured by EMFISIS. In **b**, a harmonic structure of wave power enhancement is labeled *f*_1_ (fundamental mode), *f*_2_ (second harmonic mode), and *f*_3_ (third harmonic mode). The bottom panel shows the plasma wave spectrogram for the electric field measured by EMFISIS. A white/black line delineates the local cyclotron frequency of electrons (*f*_ce_). **e**–**h** Arase observations during 03:00–11:00 UT on 12 September 2017. The format is the same as **a**–**d** except that the magnetic field data are obtained by MGF and the plasma wave spectrogram for the electric field is obtained by PWE-HFA and PWE-OFA

Figure 2a–c displays the dynamic power spectra of the magnetic field variations measured by the Electric and Magnetic Field Instrument Suite and Integrated Science (EMFISIS) instrumentation suite (Kletzing et al. 2013) onboard Probe B for 02:00–09:30 UT on 12 September 2017. We here used 1-s cadence data. The power spectra were calculated with a sliding window 1024-s (i.e., 1024 data points) wide, successively shifted by 128 s (i.e., 128 data points). No smoothing of the power spectra was performed. The magnetic field variations are expressed in local magnetic (LMG) coordinates, where the local magnetic field is calculated by the combination of the International Geomagnetic Reference Field (IGRF)-12 internal model (Thébault et al. 2015) and the Tsyganenko 1989 (T89) external model with *K*_p_ = 0 (Tsyganenko, 1989). In LMG coordinates, the parallel direction is taken to be the direction of the local field; the radial direction is perpendicular to the local field and points radially outward; and the azimuthal direction is eastward to complete the right-handed system. The magnetic field variations in the parallel component (Δ*B*_parallel_) are determined by subtracting the model field from the parallel component of the observed field. Figure 2d shows the plasma wave spectrogram of the electric field measured by Probe B/EMFISIS. White and black curves indicate the local cyclotron frequency of electrons (*f*_ce_) computed from the EMFISIS data.

In the azimuthal component (Fig. 2b), a harmonic structure of wave power enhancement can be clearly seen as labeled *f*_1_ (fundamental mode), *f*_2_ (second harmonic mode), and *f*_3_ (third harmonic mode). The wave frequencies become lower as Probe B moves closer to its apogee. In both the radial and compressional components (Fig. 2a and c) there are no such harmonic structures. This is a typical signature of the standing Alfvén wave observed in the magnetosphere. One may note that there are strong power enhancements (> 10^2^ nT^2^/Hz) during 02:30–03:15 UT in both the radial and azimuthal components. Since this wave has larger power in the radial component around 20 mHz, it may be the transverse Pc3–4 wave caused by upstream waves propagating from the magnetosheath into the inner magnetosphere (e.g., Takahashi and Anderson, 1992; Yumoto, 1986). In Fig. 2d, we find a clear emission of the upper hybrid resonance (UHR) waves. Their frequency (*f*_UHR_) showed a sudden drop at 02:30 UT and rise at 09:20 UT, which is considered the plasmapause. Between 02:30 UT and 09:20 UT, the UHR waves are sometimes overlapped by electron cyclotron harmonic emissions or (*n *+ 1/2) *f*_ce_ bands (Kurth 1982; Kurth et al. 2015).

**Fig. 3:**
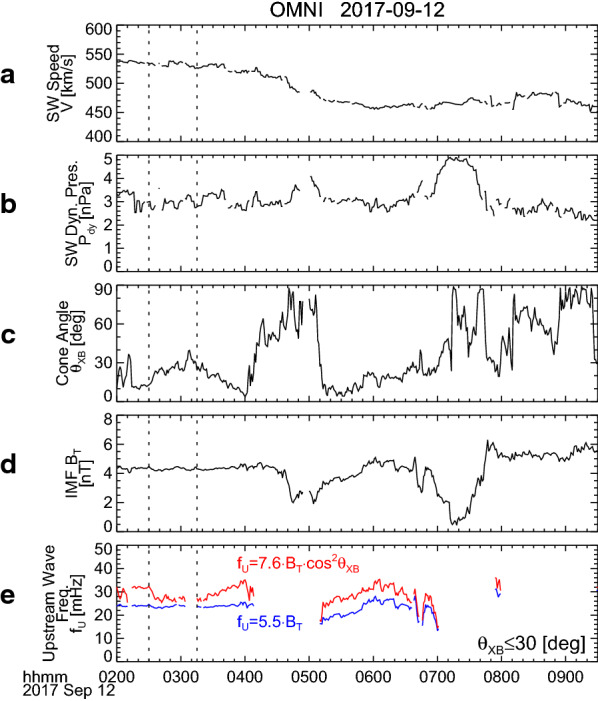
OMNI data of **a** the solar wind speed, **b** the solar wind dynamic pressure, **c** the cone angle (*θ*_XB_), and **d** the magnitude of the interplanetary magnetic field (*B*_T_) at the Earth’s bow shock nose, as well as **e** estimated frequency of upstream waves (*f*_U_) for 02:00–09:30 UT on 12 September 2017. *f*_U_ is estimated from empirical equations by Gul’elmi (1974) (blue curve) and Takahashi et al. (1984) (red curve) only when *θ*_XB_ ≤ 30°. Vertical dotted lines delineate the time interval of 02:30–03:15 UT when a transverse Pc3–4 wave with the frequency of ~ 20 mHz was observed by Probe B

Here, we test if the transverse Pc3–4 wave observed during 02:30–03:15 UT is caused by upstream waves or not. We plotted in Fig. 3a–c the solar wind speed, dynamic pressure, and cone angle at the Earth’s bow shock nose from the OMNI database for 02:00–09:30 UT on 12 September 2017. The cone angle (*θ*_XB_) is defined as *θ*_XB_ = cos^−1^(|*B*_X_|/*B*_T_), where *B*_X_ is the X component of the interplanetary magnetic field (IMF) in geocentric solar ecliptic (GSE) coordinates and *B*_T_ is the magnitude of IMF. We note that during 02:30–03:15 UT as indicated by vertical dotted lines, the solar wind speed and dynamic pressure were rather constant at 540 km/s and 3 nPa, respectively. In the same time, *θ*_XB_ had a rather small value of 10°–40° with mostly less than 30°, which is a preferable condition for excitation of upstream waves (e.g., Odera 1986; Verö 1986). Figure 3d and e indicates *B*_T_ and frequency of upstream waves (*f*_U_). *f*_U_ is estimated from empirical equations, *f*_U_ [mHz] = 5.5∙*B*_T_ [nT] (Gul’elmi 1974; blue curve) and *f*_U_ [mHz] = 7.6∙*B*_T_ [nT]∙cos^2^*θ*_XB_ (Takahashi et al. 1984; red curve) only when *θ*_XB_ ≤ 30°. The estimated values of *f*_U_ between the vertical dotted lines are 24–28 mHz, which are higher than the observed frequency (~ 20 mHz). Moreover, *θ*_XB_ was less than 30° in later time intervals (03:15–04:10 UT and 05:10–07:00 UT), but no Pc3–4 waves were observed by Probe B (Fig. 2a). These results indicate that this transverse Pc3–4 wave is not due to upstream waves. Instead, a possible explanation is the fundamental standing wave caused by the drift-bounce resonance (e.g., Dai et al. 2013; Oimatsu et al. 2018; Yamamoto et al. 2018), although we do not further discuss this possibility, because it is beyond the scope of this study.

In Fig. 3b, it should be noted the solar wind dynamic pressure showed a temporal increase up to 5 nPa for 06:50–07:50 UT, whose effect on the magnetosphere will be discussed in the later section.

Figure 2e–h shows the dynamic power spectra of the magnetic field variations and plasma wave spectrogram of the electric field observed by Arase for 03:00–11:00 UT on 12 September 2017. The magnetic field variations are measured by Magnetic Field Experiment (MGF) (Matsuoka et al. 2018). We used data averaged over the spin period (~ 8 s). In calculation of the power spectra, we used a sliding window 960-s (i.e., 120 data points) wide, successively shifted by 120 s (i.e., 15 data points). The power spectra were not smoothed. The plasma wave spectrograms below and above 20 kHz come from the onboard frequency analyzer (OFA) and the high-frequency analyzer (HFA) of the Plasma Wave Experiment (PWE), respectively (Kasahara et al. 2018; Kumamoto et al. 2018; Matsuda et al. 2018a). The magnetic field variations were rather active in both the radial and azimuthal components. In Fig. 2f, there is a signature of the fundamental mode of the standing Alfvén wave for approximately 03:30–05:00 UT, which is labeled *f*_1_. However, in the inbound path after 07:00 UT, no clear standing wave signatures can be found, because Arase was flying on the nightside (Fig. 1a) where the ionospheric conductivity is so low that the Alfvén wave cannot be reflected effectively to form the standing wave. We can identify clear UHR wave emissions in Fig. 2h. A sharp drop and rise in *f*_UHR_ appeared around 03:50 UT and 10:00 UT, respectively, which indicate that Arase traversed the plasmapause. Appearance of the electron cyclotron harmonic emissions near the UHR emissions is the same as that in the Probe B observations (Fig. 2d).

### Estimation of plasma mass density, electron number density, and average plasma mass

From the frequencies of the standing Alfvén waves and UHR waves, we estimate the plasma mass density (*ρ*) and electron number density (*n*_e_). In estimation of *ρ*, the MHD wave equation proposed by Singer et al. (1981) is numerically solved with the IGRF-12 and T89 magnetic field model for *K*_p_ = 2 and the power law model of the field-aligned distribution of *ρ* (*ρ* ∝ *r*^−α^, where *r* is the geocentric distance). Here, we used *α* = 0.5, since Takahashi et al. (2004) reported *α* = 0–1 at *L* = 4–6. The Kp index on the first half-day of 12 September 2017 was 2, 2 − , 2 + , 2. From the definition of *f*_UHR_, we can calculate *n*_e_ in cubic centimeter by $n_\text{e} ={ (f^{2}_{\text{UHR}}-f^{2}_{\text{ce}})} / {8980^2}$, where the frequencies are measured in hertz. When the UHR emission is mixed up with the electron cyclotron harmonic emissions or (*n *+ 1/2)*f*_ce_ bands, we use the frequency at which the emission is the most intense. Finally, the average plasma mass *M* can be estimated by *M* ~ *ρ*/*n*_e_. More detailed description about estimation of *ρ*, *n*_e_, and *M* can be found in papers by Nosé et al. (2011, 2015).

**Fig. 4:**
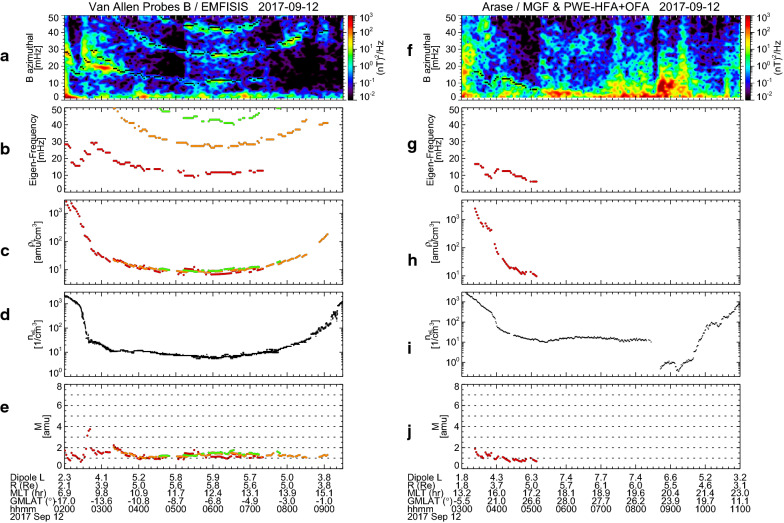
Van Allen Probe B observations of **a** the dynamic power spectrum of the magnetic field variations in the azimuthal component with selected peak frequency indicated by black dots, **b** the selected peak frequency, **c** mass density at the satellite position, **d** electron density at the satellite position, and **e** average plasma mass, during 02:00–09:30 UT on 12 September 2017. In **b**–**e**, colors indicate results of different eigen-frequencies, that is, fundamental (*f*_1_): red, second harmonic (*f*_2_): orange, and third harmonic (*f*_3_): green. **f**–**j** Same as in **a**–**e** but for the Arase observations during 03:00–11:00 UT on 12 September 2017

Figure 4 compiles the results for Probe B (left panels) and Arase (right panels). Figure 4a shows the dynamic power spectrum of *B*_azimuthal_ that is identical to Fig. 2a, except that the standing wave frequencies (*f*_*p*_) were selected by a procedure similar to those used by Nosé et al. (2011, 2015) and represented with black dots. In the selection procedure, first, the following criteria was applied to the power spectra: (1) *P*(*f*_*p*_)∕*P*(*f*_*p*_ − Δ*f*) > 1.05 and *P*(*f*_*p*_ −Δ*f*)∕*P*(*f*_*p*_ − 2Δ*f*) > 1.05, (2) *P*(*f*_*p*_)∕*P*(*f*_*p*_ + Δ*f*) > 1.05 and *P*(*f*_*p*_ + Δ*f*)∕*P*(*f*_*p*_ + 2Δ*f*) > 1.05, and (3) *P*(*f*_*p*_) ≥ 10^−0.5^ nT^2^/Hz, where *P*(*f*) is the wave power at frequency *f* and Δ*f* is the frequency resolution (Δ*f* = 0.98 mHz). Second, the selected *f*_*p*_ was plotted and visually compared with the dynamic power spectra, and we removed some data points of *f*_*p*_ if they do not match the harmonic structure. Probe B was moving from *L* = 3.3 to *L* = 4.7 during 02:30–03:30 UT and had an average speed of 0.023 min^−1^ in *L*. Since the power spectrum is calculated with a 1024-s sliding window shifted by 128 s, the black dots have the spatial coverage and resolution of ~ 0.4 and ~ 0.05 in *L*, respectively, around *L* = 3.3–4.7. In Fig. 4b, the selected frequencies are replotted with identification of harmonics in colors (*f*_1_: red, *f*_2_: orange, and *f*_3_: green). From these frequencies, we estimate *ρ* at the satellite position (*ρ*_L_) as shown in Fig. 4c. Different harmonics give the similar values of *ρ*_L_, which justifies our estimation of *ρ*. Figure 4d shows *n*_e_ at the satellite position (*n*_eL_) estimated from the UHR emission. The clear plasmapause is found around 02:30 UT, when Probe B was located at *L* = 3.3–3.6 and MLT = 9.0 h. There is also the plasmapause around 09:20 UT corresponding to *L* ~ 3.3 and MLT = 15.7 h, although it is not as clear as that of ~ 02:30 UT. Outside the plasmapause, *n*_eL_ is larger than ~ 10 cm^−3^, which indicates that Probe B was flying in a partially refilling region. We consider it is possible because the event was found during the recovery phase of the magnetic storm (Fig. 1b). From *ρ*_L_ and *n*_eL_, we calculate *M* as shown in Fig. 4e. Almost all of the time interval, *M* is staying at 1.0–1.5 amu, indicating that the background plasma was mostly composed of protons. There are some data points well below 1 amu (~ 0.6 amu) at 02:00–02:30 UT (*L* < 3.3 and geomagnetic latitude (GMLAT) < − 15.5°). We suppose that this may be due to an inadequate value of α. A larger value of α gives a larger estimation value of *M* at|GMLAT| ≥ 12° (Takahashi et al. 2006); if α > 0.5 is used in the calculation, that will result in *M* ~ 1 amu for these data points. We notice that *M* is enhanced up to 3–4 amu at 02:40 UT, when Probe B was located just outside the plasmapause at *L* ~ 3.6 and MLT ~ 9.2 h. Such large value of *M* can be created by 70–100% He^+^ with 30–0% H^+^, but He^+^ dominance is unlikely in the magnetosphere; thus, we should consider whether O^+^ ions can explain the observation. If we assume a plasma including H^+^ and the same amount of He^+^ and O^+^, *M* = 3.5 amu can be produced by 72% H^+^, 14% He^+^, and 14% O^+^. (As shown later, the more realistic composition is estimated as 80.6% H^+^, 3.4% He^+^, and 16.0% O^+^.) Therefore, we suppose that the oxygen torus is identified just outside the plasmapause at *L* ~ 3.6 on the morning side by Probe B. It should be noted, however, that Probe B flying near the plasmapause on the afternoon side around 09:00 UT did not seem to find a similar enhancement of *M*.

The same data processing is conducted for the Arase data and the results are shown in Fig. 4f–j in the same format of Fig. 4a–e. Note that the selection procedure for *f*_*p*_ has Δ*f* = 1.04 mHz in Fig. 4f, because the power spectra were calculated for the 960-s data segment. During 04:00–05:00 UT, Arase had an average speed of 0.033 min^−1^ in *L*, resulting that the black dots in Fig. 4f have the spatial coverage and resolution of ~ 0.53 and ~ 0.067 in *L*, respectively. As seen in Fig. 4i, Arase observed the plasmapause at 04:00 UT corresponding to *L* ~ 4.3 and MLT ~ 16.0 h, and then flew through a partially refilling region (*n*_eL_ ~ 10–30 cm^−3^) until 08:30 UT. For 08:40–09:40 UT, *n*_e_ suddenly decreases to less than 1 cm^−3^, which is a signature of the plasmatrough. After 09:40 UT, *n*_eL_ shows a gradual increase to ~ 200 cm^−3^ within ~ 1 h, indicating encounter with the plasmapause on the nightside. In Fig. 4j, *M* can be estimated only in the afternoon sector, where it is generally 1.0–1.5 amu and the background plasma is mostly protons. No distinct enhancement of *M* is found outside the plasmapause, which is consistent with the Probe B observation on the afternoon side (Fig. 4e). We have no information about *M* on the nightside for this event, since there are no clear standing wave signatures. The possibility of higher *M* near the nightside plasmapause can not be ruled out. Instruments that can measure low energy (< 1 eV) ion flux, such as DE-1/RIMS, are needed to examine the background ion composition on the nightside. Or, a new technique to estimate O^+^ ion concentration from ion temperature information proposed by Goldstein et al. (2019) may be useful.

### Electromagnetic ion cyclotron waves in oxygen torus

**Fig. 5:**
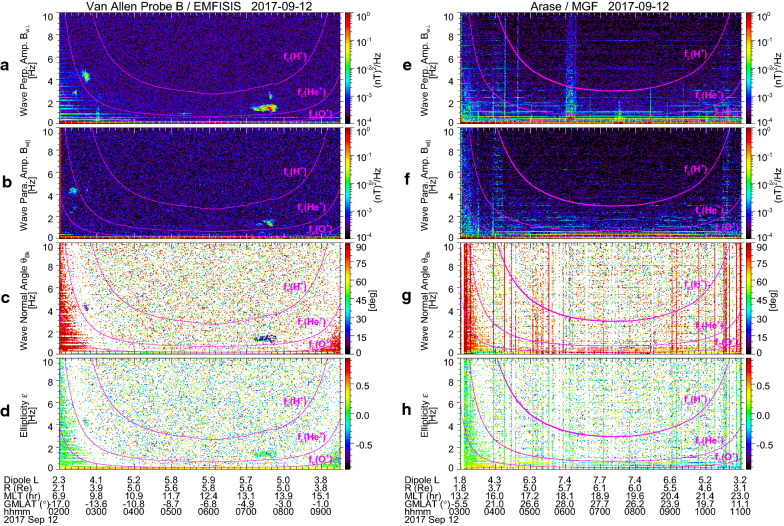
Van Allen Probe B/EMFISIS observations of (**a**, **b**) the dynamic power spectra of wave amplitudes in the directions perpendicular and parallel to the local magnetic field, **c** wave normal angle with respect to the magnetic field, and **d** wave ellipticity, from 02:00 UT to 09:30 UT on 12 September 2017. Positive (negative) values of the wave ellipticity indicate right-handed (left-handed) polarization. Values of the wave normal angle and the ellipticity are shown only when degree of polarization is greater than or equal to 0.8. Magenta lines indicate local cyclotron frequencies of H^+^, He^+^, and O^+^ ions. **e**–**h** Same as in **a**–**d** but for the Arase/MGF observations during 03:00–11:00 UT on 12 September 2017

**Fig. 6:**
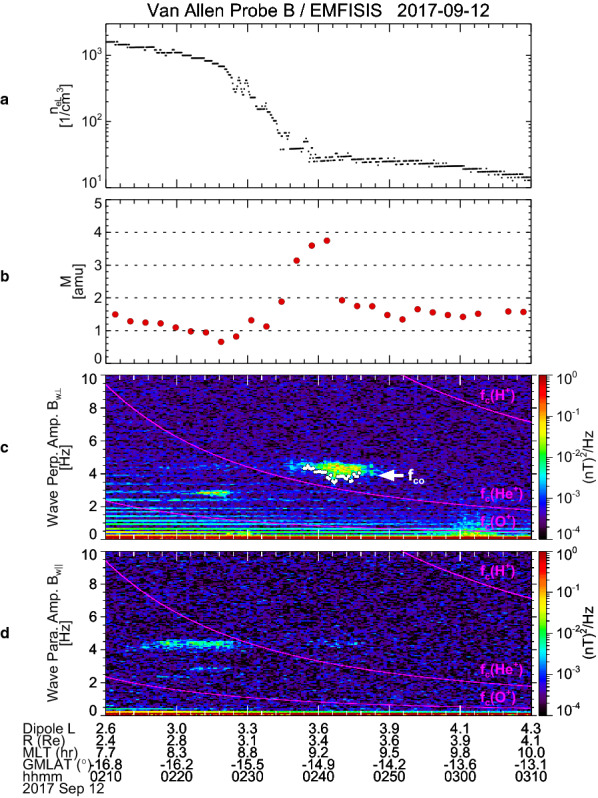
Van Allen Probe B/EMFISIS observations of **a** electron density at the satellite position, **b** average plasma mass, and **c**, **d** the dynamic power spectra of wave amplitudes in the directions perpendicular/parallel to the local magnetic field, during 02:10–03:10 UT on 12 September 2017. Data are the same as **d**, **e**, 5**a**, and **b**. White dots indicate the lower cutoff frequency (*f*_co_) of the emission of an electromagnetic ion cyclotron wave

We examine whether the local enhancement of O^+^ density is related to EMIC occurrence. In Fig. 5a–d, from top to bottom, the dynamic power spectra of wave amplitudes in the directions perpendicular (*B*_w⊥_) and parallel (*B*_w||_) to the local magnetic field, wave normal angle with respect to the magnetic field (*θ*_Bk_), and wave ellipticity (*ε*) (Means, 1972; Samson and Olson, 1980) are displayed for Probe B from 02:00 UT to 09:30 UT. Positive (negative) values of *ε* indicate right-handed (left-handed) polarization. Values of *θ*_Bk_ and *ε* are shown only when degree of polarization is greater than or equal to 0.8 (Samson and Olson, 1980). Magenta lines delineate local cyclotron frequencies of H^+^, He^+^, and O^+^ ions. We find from Fig. 5a that distinct wave activities appear in *B*_w⊥_ around 02:40–02:50 UT and 07:10–07:50 UT in the frequency range of 1–5 Hz. They have the general EMIC characteristics such that the wave frequency depends on the ion cyclotron frequency, the wave propagation direction is nearly parallel to the magnetic field (*θ*_Bk_ ~ 0°), and the wave amplitude is larger in *B*_w⊥_ than *B*_w||_. It is also noted in Fig. 5d that *ε* of these waves is close to 0; that is, they have nearly linear polarization. This result is consistent with a previous result by Anderson et al. (1992) that an EMIC wave on the dawn side at ~ 5° GMLAT occurred with a predominantly linear polarization. A statistical study by Min et al. (2012) showed that the H^+^ band EMIC waves at dawn are weakly left-handed polarized near the equator and become linearly polarized with increasing latitude up to |GMALT| ~ 15°. Fraser and Nguyen (2001) derived the similar conclusion from a statistical analysis of the CREES satellite data. The *B*_w⊥_ waves around 02:40–02:50 UT and 07:10–07:50 UT were observed when Probe B was located at approximately −15° GMLAT and −4° GMLAT, respectively. We, therefore, consider that they are H^+^ band EMIC waves observed off the equator. One may note that there are also wave activities around 02:20–02:30 UT in *B*_w⊥_ at ~ 3 Hz and *B*_w||_ at ~ 4.5 Hz. Their frequencies appear almost constant. (Expanded plots demonstrating the constant frequencies are provided in Fig. 6.) This frequency characteristic is similar to that of propagating EMIC waves observed at *L* = 2–5 by the Akebono (Sakaguchi et al. 2013) and Arase (Matsuda et al. 2018b) satellites. However, we do not further focus on them, because they have much smaller wave power than the waves mentioned above (the *B*_w⊥_ waves around 02:40–02:50 UT and 07:10–07:50 UT), they only appear for a short time, and they seem to show no clear values of *θ*_Bk_ and *ε* (i.e., degree of polarization less than 0.8). Moreover, the wave activity in *B*_w||_ at ~ 4.5 Hz is different from the fact that EMIC waves are typically transverse.

As noted in Fig. 3, the solar wind dynamic pressure was enhanced from 3 nPa to 5 nPa during 06:50–07:50 UT, which almost corresponds to the time interval of the latter EMIC appearance. We suppose that the enhancement of the solar wind dynamic pressure causes compression of the dayside magnetosphere and local betatron acceleration of ions. Then the ions accelerated in the perpendicular direction would have a pitch angle distribution with a larger anisotropy to excite the EMIC wave at 07:10–07:50 UT (Anderson and Hamilton, 1993; Anderson et al. 1996). However, for the former EMIC wave that appeared at approximately 4.5 Hz around 02:40–02:50 UT, no enhancement of the solar wind dynamic pressure occurred (Fig. 3). Instead, this EMIC wave appeared when *M* was enhanced up to 3–4 amu, that is, around 02:40 UT as shown in Fig. 4e. To confirm the coincidence of the EMIC wave and the oxygen torus, we plotted *M*, the dynamic power spectra of *B*_w⊥_ and *B*_w||_ along with *n*_eL_ for 02:10–03:10 UT in Fig. 6. It is clearly shown that the EMIC wave appeared just outside the plasmapause. The interval of the EMIC wave overlaps the interval when *M* is larger than approximately 2 amu, but it seems to slightly extend toward larger *L*. This may be due to an oblique propagation of the EMIC wave from the excitation region near the equatorial plane to the Probe B location (i.e., approximately −15° GMLAT). However, the general coincidence between the EMIC wave and the oxygen torus implies that the composition of the background plasma has an effect on excitation of EMIC waves.

Probe B encountered the plasmapause in the afternoon around 09:20 UT and no oxygen torus was found there, as seen in Fig. 4d and e. There is correspondingly no EMIC wave activity around 09:20 UT in Fig. 5a and b. The similar features are noticed in the Arase observations. Figure 5e–h displays the same as Fig. 5a–d, except for the Arase/MGF data. When Arase was near the plasmapause in the afternoon around 04:00 UT, *M* stayed at 1.0–1.5 amu, as seen in Fig. 4i and j. No EMIC wave activity can be identified around 04:00 UT in Fig. 5e and f. These results also imply a close relation between the background plasma composition and the EMIC wave excitation.

## Discussion

### Longitudinal structure of oxygen torus

It is found from Fig. 4d and e that Probe B observed a local enhancement of *M* = 3–4 amu just outside the plasmapause at *L* ~ 3.6 and MLT ~ 9.2 h in its outbound path, but no similar enhancement was identified near the plasmapause along the inbound path in the afternoon. Arase also did not observe an enhancement of *M* near the plasmapause in the afternoon (Fig. 4i and j). These simultaneous observations by Probe B and Arase on 12 September 2017 demonstrates that the oxygen torus is not axisymmetric but skewed toward the morning side. This is consistent with the result reported by Nosé et al. (2018) for the 24 April 2017 event, in which the mutual longitudinal position of the satellites was reverse, that is, Arase in the morning and Van Allen Probe in the afternoon. We therefore think that the oxygen torus usually has a crescent shape centered on the morning side, as proposed by Nosé et al. (2015, 2018).

### Ion composition of oxygen torus

In “Estimation of plasma mass density, electron number density, and average plasma mass” section, the simple assumption of identical number densities of He^+^ and O^+^ gives an estimation of the ion composition to be 72% H^+^, 14% He^+^, and 14% O^+^. However, the EMIC waves in the oxygen torus provide us a clue to estimate the ion composition more precisely. The similar approach has been applied for EMIC waves and equatorial noise emissions observed by Arase (Miyoshi et al. 2019). From Fig. 6, we determined the lower cutoff frequency (*f*_co_) of the EMIC wave with a criterion *P*(*f *≥* f*_co_) ≥ 3 × 10^−3^ nT^2^/Hz, where *P*(*f*) is the wave power at frequency *f*. White dots show the results of determination of *f*_co_. It is found that the average value of *f*_co_ during 02:38–02:46 UT is 3.95 Hz. For a three-component plasma consisting of H^+^, He^+^, and O^+^ ions with fractions of $p_{{{\text{H}}^{ + } }}$, $p_{{{\text{He}}^{ + } }}$, and $p_{{{\text{O}}^{ + } }}$, the theoretical equation of the cutoff frequency for the H^+^ band EMIC waves is given as

1 $$ f_{\text{co}} = \frac{{f_{{{\text{cH}}^{ + } }} }}{32}\left\{ { - 16p_{{{\text{H}}^{ + } }} - 4p_{{{\text{He}}^{ + } }} - p_{{{\text{O}}^{ + } }} + 21 + \sqrt {\left( {16p_{{{\text{H}}^{ + } }} + 4p_{{{\text{He}}^{ + } }} + p_{{{\text{O}}^{ + } }} - 21} \right)^{2} + 16\left( {20p_{{{\text{H}}^{ + } }} + 17p_{{{\text{He}}^{ + } }} + 5p_{{{\text{O}}^{ + } }} - 21} \right)} } \right\} , $$

where $f_{{{\text{cH}}^{ + } }}$ is the cyclotron frequency of H^+^ (Min et al. 2015). The average value of $f_{{{\text{cH}}^{ + } }}$ during 02:38–02:46 UT is found to be 12.6 Hz from Probe B observations. Other two equations with respect to $p_{{{\text{H}}^{ + } }}$, $p_{{{\text{He}}^{ + } }}$, and $p_{{{\text{O}}^{ + } }}$ are derived as follows:

2 $$ p_{{{\text{H}}^{ + } }} + p_{{{\text{He}}^{ + } }} + p_{{{\text{O}}^{ + } }} = 1 , $$

3 $$ p_{{{\text{H}}^{ + } }} + 4p_{{{\text{He}}^{ + } }} + 16p_{{{\text{O}}^{ + } }} = M . $$

Equation (2) comes from the characteristics of the fractions and Eq. (3) represents definition of the average plasma mass. Here we take *M* = 3.5 amu on the basis of Probe B observations (Figs. 4e and 6b). Then, the simultaneous Eqs. (1)–(3) can be solved, and we obtain $p_{{{\text{H}}^{ + } }}$ = 0.806, $p_{{{\text{He}}^{ + } }}$ = 0.034, and $p_{{{\text{O}}^{ + } }}$ = 0.160. We conclude that the oxygen torus for this event is composed of 80.6% H^+^, 3.4% He^+^, and 16.0% O^+^, and the fraction of O^+^ is much higher than that of He^+^ ($p_{{{\text{O}}^{ + } }}$ / $p_{{{\text{He}}^{ + } }}$ = 4.7).

The solution of $p_{{{\text{H}}^{ + } }}$, $p_{{{\text{He}}^{ + } }}$, and $p_{{{\text{O}}^{ + } }}$ depends on values of *f*_co_ and *M* that are estimated from the Probe B observations, where uncertainties should be considered. We also solved the simultaneous Eqs. (1)–(3) when *f*_co_ and *M* are altered in reasonable ranges. In Fig. 6c, the background level of the power in *B*_w⊥_ is mostly less than 5 × 10^−4^ nT^2^/Hz. If the criterion *P*(*f *≥* f*_co_) ≥ 5 × 10^−4^ nT^2^/Hz is adopted, we have *f*_co_ = 3.76 Hz as the average value for 02:38–02:46 UT, instead of *f*_co_ = 3.95 Hz. In Fig. 4e, the oxygen torus was identified as *M* = 3–4 amu, and we take two alternative values of *M* = 3.0 and 4.0 amu. Table 1 compiles the results of solution for six different cases of *f*_co_ and *M* (i.e., combination of *f*_co_ = 3.76 and 3.95 Hz and *M* = 3.0, 3.5, and 4.0 amu). It is found that $p_{{{\text{O}}^{ + } }}$ increases as *f*_co_ decreases and *M* increases, but it is opposite for $p_{{{\text{He}}^{ + } }}$. These six cases give $p_{{{\text{H}}^{ + } }}$ = 0.78–0.84, $p_{{{\text{He}}^{ + } }}$ = 0.01–0.05, and $p_{{{\text{O}}^{ + } }}$ = 0.12–0.20. Thus, the uncertainties of the ion composition are ± 2–4%. The ion composition of the oxygen torus in this event is very different from the values during the main phase of a large (Dst_min_ = − 176 nT) magnetic storm reported by Grew et al. (2007), that is, $p_{{{\text{H}}^{ + } }}$ = 0.05–0.47, $p_{{{\text{He}}^{ + } }}$ = 0.07–0.15, and $p_{{{\text{O}}^{ + } }}$ = 0.46–0.81.

**Table 1:** Solution of $p_{{{\text{H}}^{ + } }}$, $p_{{{\text{He}}^{ + } }}$, and $p_{{{\text{O}}^{ + } }}$ for different values of *f*_co_ and *M*

*f*_co_ (Hz)	*M* (amu)	$p_{{{\text{H}}^{ + } }}$	$p_{{{\text{He}}^{ + } }}$	$p_{{{\text{O}}^{ + } }}$
3.76	3.0	0.841	0.032	0.127
3.76	3.5	0.815	0.023	0.162
3.76	4.0	0.789	0.014	0.197
3.95	3.0	0.830	0.045	0.124
3.95	3.5	0.806	0.034	0.160
3.95	4.0	0.782	0.023	0.195

### Effect of O^+^ ions on growth rate of electromagnetic ion cyclotron waves

#### Growth rate calculation for 3 cases

Probe B found the coincidence of the oxygen torus and the H^+^ band EMIC wave around 02:40 UT (Fig. 6). Then, we calculate the growth rate of the H^+^ band EMIC wave, according to the linearized dispersion relation for EMIC waves derived by Gomberoff and Neira (1983) and Kozyra et al. (1984). The Probe B observations during 02:38–02:46 UT show that the cold ion density (*n*_ci_) and the magnitude of the background magnetic field (*B*_0_) at −15° GMLAT are 29.8 cm^−3^ and 825 nT, respectively. Since the EMIC waves are likely to be excited at the equator, we calculated *n*_ci_ and *B*_0_ at the equator, assuming the power law model of the field-aligned distribution of plasma (∝ *r*^−0.5^) and the dipole field. The equatorial values are *n*_ci_ = 28.8 cm^−3^ and *B*_0_ = 611 nT. We consider three cases of the ion composition of the cold plasma: (1) (H^+^:He^+^:O^+^) = (80.6:3.4:16.0) for a case of the oxygen torus, (2) (H^+^:He^+^:O^+^) = (80.6:19.4:0.0) for a case of no O^+^ ions, and (3) (H^+^:He^+^:O^+^) = (67.8:19.4:12.8) for a case of the same *M* with fewer O^+^ ions. The first case is based on the Probe B result discussed in “Ion composition of oxygen torus” section; and the second case assumes the identical fraction of H^+^ ions (80.6%) with He^+^ ions for all the rest (19.4%), resulting in smaller *M* (~ 1.6 amu). The third case keeps the same *M* as the case of the oxygen torus (3.5 amu), but includes a small amount of O^+^ ions. A free energy source for EMIC excitation is assumed to be supplied by warm proton plasma. From results by Lui and Hamilton (1992) and Wing and Newell (1998), we take the density (*n*_wp_) of 2 cm^−3^, the energy parallel and perpendicular to the magnetic field ($W_{\parallel}$ and *W*_⊥_) of 15 keV and 30 keV, respectively, which give anisotropy (*A* = *W*_⊥_/$W_{\parallel}$ − 1) of 1.0.

**Fig. 7:**
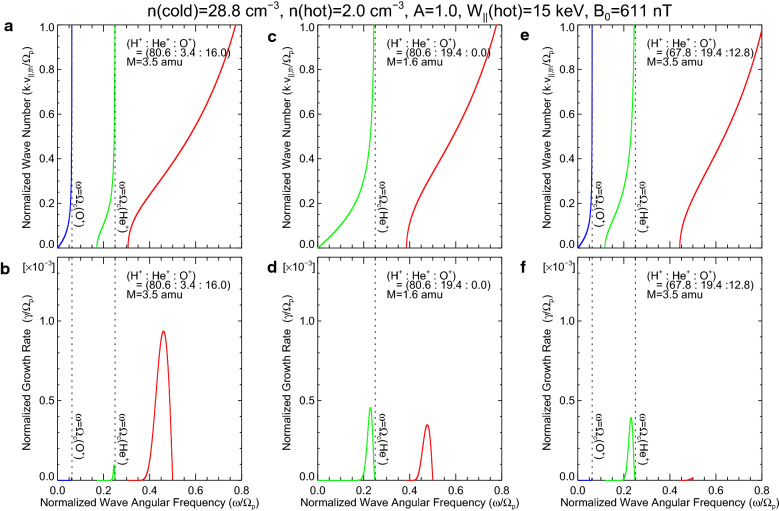
Solution of the linearized dispersion relation for electromagnetic ion cyclotron (EMIC) waves. **a** Wave number and **b** growth rate as a function of wave angular frequency for the oxygen torus case, that is, ion composition of (H^+^:He^+^:O^+^) = (80.6:3.4:16.0). Red, green, and blue curves present the branch of the H^+^, He^+^, and O^+^ band EMIC waves, respectively. Parameters used in the calculation are displayed in the top of panels. **c**, **d** Same as in **a**, **b** except for the no O^+^ ion case, that is, the ion composition of (H^+^:He^+^:O^+^) = (80.6:19.4:0.0). **e**, **f** Same as in **a** and **b** except for *M* identical to the oxygen torus case (3.5 amu) with less O^+^ ions, that is, the ion composition of (H^+^:He^+^:O^+^) = (67.8:19.4:12.8)

Figures 7a and b are solutions of the linearized dispersion relation for the oxygen torus case (case 1). From the relation between the wave number and the wave angular frequency (i.e., *k*-*ω* diagram) shown in Fig. 7a, we found that there are three branches of EMIC waves (red, green, and blue curves for the H^+^, He^+^, and O^+^ bands, respectively) and the H^+^ band EMIC wave can be present at *ω*/*ω*_p_ ≥ 0.31, where *ω*_p_ is the angular cyclotron frequency of H^+^. The growth rate (γ) as a function of *ω* is given in Fig. 7b. It is found that the growth rate of the H^+^ band branch is positive for *ω*/*ω*_p_ = 0.31–0.50 and has the largest peak at *ω*/*ω*_p_ ~ 0.46. From *B*_0_ = 611 nT at the equator, we derive *ω*_p_ ~ 58.5 rad/s, resulting that the frequency giving the largest growth rate is 0.46 × 58.5/(2*π*) ~ 4.3 Hz. This is consistent with the Probe B observation of the H^+^ band EMIC around 4.5 Hz.

Figure 7c and d displays the calculation results for the no O^+^ case (case 2). Since O^+^ ions are not included, there are only two branches and the H^+^ band branch (red curve) can be present at *ω*/*ω*_p_ ≥ 0.39 (Fig. 7c). The growth rate of the H^+^ band branch is positive for *ω*/*ω*_p_ = 0.39–0.50 and has a peak at *ω*/*ω*_p_ ~ 0.48 (Fig. 7d). Results for the same *M* with less O^+^ ions (case 3) are displayed in Fig. 7e and f. The H^+^ band branch (red curve) is identified at *ω*/*ω*_p_ ≥ 0.44, but its growth rate is almost zero.

The comparison of Fig. 7b and d reveals that the peak value of the growth rate is about 2.8 times larger for the oxygen torus case (case 1, γ/*ω*_p_ ~ 0.94 × 10^−3^) than for the no O^+^ case (case 2, γ/*ω*_p_ ~ 0.34 × 10^−3^). In Fig. 7f, we find that the growth rate for case 3 is negligible in comparison with that for case 1.

Here, it is worthwhile briefly mentioning the O^+^ band EMIC waves. The present results for cases 1 and 3 show that there is a branch of the O^+^ band EMIC waves (blue curve) at *ω*/*ω*_p_ ≤ 0.0625 (Fig. 7a and e), but its growth rate is nearly zero (Fig. 7b and f). However, Yu et al. (2015) found 18 events of O^+^ band EMIC waves at MLT = 03–13 h and 19–20 h, which is consistent with the longitudinal extent of high occurrence rate of the oxygen torus. We suppose that such O^+^ band EMIC waves were excited when the plasma condition was different from the present cases, for example, warm plasma with different energy or anisotropy, background cold plasma with different density or ion composition, and so on.

#### Growth rate of H^+^ band EMIC waves for various $p_{{{\text{H}}^{ + } }}$, $p_{{{\text{He}}^{ + } }}$, and $p_{{{\text{O}}^{ + } }}$

**Fig. 8:**
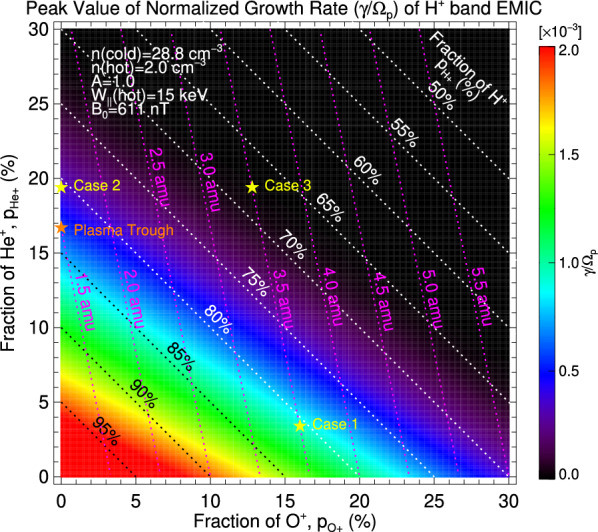
Peak values of γ/*ω*_p_ as a function of $p_{{{\text{He}}^{ + } }}$ and $p_{{{\text{O}}^{ + } }}$, using the same parameters as Fig. 7 except for the ion composition. Color scale indicates γ/*ω*_p_. White and black dotted lines delineate $p_{{{\text{H}}^{ + } }}$ from 50% to 95% at 5% intervals. Magenta dotted lines show *M* from 1.5 amu to 5.5 amu at 0.5 amu intervals. Yellow stars indicate the peak values of γ/*ω*_p_ for cases 1–3 (Fig. 7b, d, and f), while an orange star shows a value for the plasma trough that consists of H^+^ and He^+^ ions and has *M* = 1.5 amu

To understand the effect of the ion composition on the growth rate of the H^+^ band EMIC waves, we calculated peak values of γ/*ω*_p_ as a function of $p_{{{\text{He}}^{ + } }}$ and $p_{{{\text{O}}^{ + } }}$, using the same parameters as Fig. 7 except for the ion composition. Results are shown in Fig. 8, where the color scale indicates γ/*ω*_p_. Since the 3-component plasma is assumed, $p_{{{\text{H}}^{ + } }}$ is determined when $p_{{{\text{He}}^{ + } }}$ and $p_{{{\text{O}}^{ + } }}$ are given; and $p_{{{\text{H}}^{ + } }}$ from 50 to 95% at 5% intervals are delineated with white and black dotted lines. Once $p_{{{\text{H}}^{ + } }}$, $p_{{{\text{He}}^{ + } }}$, and $p_{{{\text{O}}^{ + } }}$ are determined, we can calculate *M*, which is plotted with magenta dotted lines for values from 1.5 amu to 5.5 amu at 0.5 amu intervals. The peak values of γ/*ω*_p_ for cases 1–3 are indicated with yellow stars.

It is revealed from Fig. 8 that increases of both $p_{{{\text{He}}^{ + } }}$ and $p_{{{\text{O}}^{ + } }}$ (i.e., decrease of $p_{{{\text{H}}^{ + } }}$) generally stabilize the H^+^ band EMIC waves, which is consistent with the result by Mace et al. (2011). This stabilizing effect is stronger for He^+^ ions than O^+^ ions. When a constraint of constant $p_{{{\text{H}}^{ + } }}$ is imposed, as can be seen along the white or black dotted lines, γ/*ω*_p_ becomes larger as $p_{{{\text{O}}^{ + } }}$ becomes larger (correspondingly, $p_{{{\text{He}}^{ + } }}$ becomes smaller). This characteristic is reflected in the comparison between case 1 and case 2. We also find that under the constraint of constant *M*, that is, along the magenta lines, γ/*ω*_p_ becomes larger as $p_{{{\text{O}}^{ + } }}$ becomes larger (correspondingly, $p_{{{\text{He}}^{ + } }}$ becomes smaller and $p_{{{\text{H}}^{ + } }}$ becomes larger). The comparison between case 1 and case 3 represents this general characteristic. These results can be interpreted by a combination of the stronger stabilizing effect of He^+^ ions and the decrease of $p_{{{\text{He}}^{ + } }}$. We therefore conclude that, when $p_{{{\text{H}}^{ + } }}$ or *M* is not varied, the denser O^+^ ions, which are naturally accompanied by the more tenuous He^+^ ions, enhance the growth rate of the H^+^ band EMIC waves. (Min et al. (2015) found a similar effect of $p_{{{\text{O}}^{ + } }}$ on the growth rate of the O^+^ band EMIC waves.)

The oxygen torus of the 12 September 2017 event has *M* ~ 3.5 amu, but its neighboring plasma in the plasma trough and the plasmasphere has *M* ~ 1.5 amu (Fig. 6b). Assuming that this light plasma in the plasma trough consists of H^+^ and He^+^ ions, that is, (H^+^:He^+^:O^+^) = (83.3:16.7:0.0), we find from Fig. 8 that γ/*ω*_p_ is 0.56 × 10^−3^ as indicated by an orange star. This growth rate is smaller than that in the oxygen torus (case 1). In the plasmasphere, the equatorial values of *n*_ci_ and *B*_0_ are larger than those in the oxygen torus. Taking *n*_ci_ = 1000 cm^−3^, *B*_0_ = 2000 nT, and *M* = 1.5 amu, we obtain γ/*ω*_p_ ~ 0.091 × 10^−3^ from the similar computation, which is also smaller than that in the oxygen torus. Thus, we suppose that the oxygen torus in the inner magnetosphere can lead to larger γ/*ω*_p_ of the H^+^ band EMIC waves than the adjacent region in the plasma trough and the plasmasphere. This is consistent with the simultaneous observations of the oxygen torus and the EMIC wave in Fig. 6b and c.

### Partial densities of H^+^, He^+^, and O^+^ ions from HOPE instrument

The Helium, Oxygen, Proton, and Electron (HOPE) mass spectrometer carried by Probe B is designed to measure ion fluxes in the energy range from 1 eV to 50 keV with mass and charge state information (Funsten et al. 2013). Using the HOPE data, we calculate partial densities of H^+^, He^+^, and O^+^ ions over 1 eV to 50 keV to compare with the present results about the oxygen torus.

**Fig. 9:**
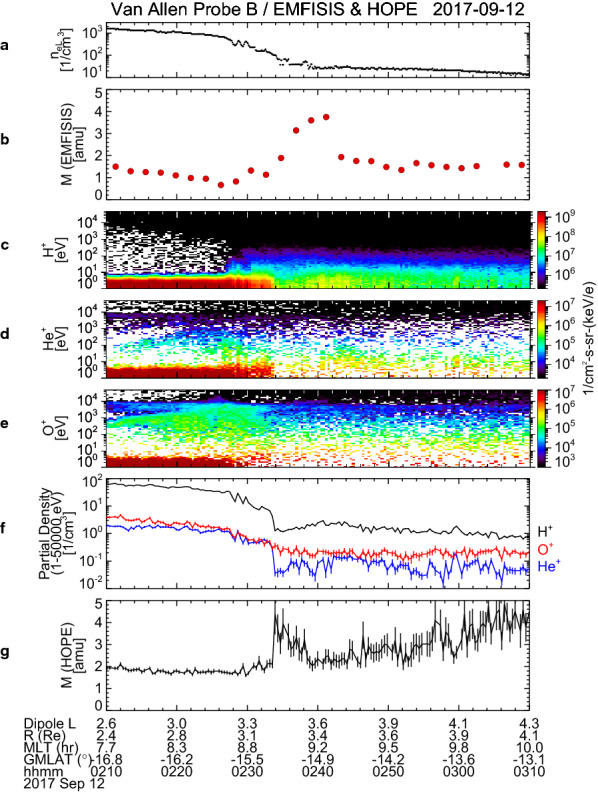
Van Allen Probe B/EMFISIS observations of **a** electron density at the satellite position and **b** average plasma mass for 02:10–03:10 UT on 12 September 2017, which are the identical to Fig. 6a and b. Van Allen Probe B/HOPE observations of **c**–**e** the energy-time diagrams of H^+^, He^+^, and O^+^ ions, **f** partial densities of H^+^ (black), He^+^ (blue), and O^+^ (red) ions calculated from ion fluxes over the energy range of 1 eV–50 keV, and **g** average plasma mass calculate from the partial densities, for 02:10–03:10 UT

Figure 9a and b displays *n*_eL_ and *M* for 02:10–03:10 UT, which are identical to Fig. 6a and b. A gradual decrease of *n*_eL_ (i.e., the plasmapause) is identified around 02:25–02:35 UT and the enhancement of *M* (i.e., the oxygen torus) is found just outside the plasmapause. The HOPE data are shown in Fig. 9c–g: from top to bottom, the energy-time diagrams of H^+^, He^+^, and O^+^ ions; their partial densities ($n^{\prime}_{{{\text{H}}^{ + } }}$, $n^{\prime}_{{{\text{He}}^{ + } }}$, and $n^{\prime}_{{{\text{O}}^{ + } }}$); and the average plasma mass calculated from the partial densities (*M′*), which is expressed as

4 $$ M^{\prime} = {{(n^{\prime}_{{{\text{H}}^{ + } }} + 4n^{\prime}_{{{\text{He}}^{ + } }} + 16n^{\prime}_{{{\text{O}}^{ + } }} )} \mathord{\left/ {\vphantom {{(n^{\prime}_{{{\text{H}}^{ + } }} + 4n^{\prime}_{{{\text{He}}^{ + } }} + 16n^{\prime}_{{{\text{O}}^{ + } }} )} {\left( {n^{\prime}_{{{\text{H}}^{ + } }} + n^{\prime}_{{{\text{He}}^{ + } }} + n^{\prime}_{{{\text{O}}^{ + } }} } \right)}}} \right. \kern-0pt} {\left( {n^{\prime}_{{{\text{H}}^{ + } }} + n^{\prime}_{{{\text{He}}^{ + } }} + n^{\prime}_{{{\text{O}}^{ + } }} } \right)}} $$

The energy-time diagrams (Fig. 8c–e) indicate that the low energy fluxes of all ion species are not reduced by positive charging of the satellite which is sometimes created by the emissions of photoelectrons from the satellite surface. This makes it possible for us to calculate the density moment over the whole energy range. However, it should be noted that the calculated density is partial, because we can expect that there are still large ion fluxes below 1 eV. This can be confirmed by comparing Fig. 9a and Fig. 9f. The total value of $n^{\prime}_{{{\text{H}}^{ + } }}$, $n^{\prime}_{{{\text{He}}^{ + } }}$, and $n'_{{{\text{O}}^{ + } }}$ is in the order of 10^2^ cm^−3^ before 02:30 UT (10^0^ cm^−3^ after 02:40 UT), while *n*_eL_ is in the order of 10^3^ cm^−3^ before 02:30 UT (10^1^ cm^−3^ after 02:40 UT). Assuming the quasi-neutrality of plasma, we consider that the partial densities shown in Fig. 9f represent only 10% of the true density. Nevertheless, it is worth pointing out that the behavior of $n'_{{{\text{O}}^{ + } }}$ near the plasmapause is different from those of $n^{\prime}_{{{\text{H}}^{ + } }}$ and $n^{\prime}_{{{\text{He}}^{ + } }}$. We note that $n'_{{{\text{O}}^{ + } }}$ decreases monotonically between 02:25 and 02:35 UT, while $n^{\prime}_{{{\text{H}}^{ + } }}$ and $n^{\prime}_{{{\text{He}}^{ + } }}$ show a decrease after 02:25 UT followed by a sudden drop around 02:34 UT. This implies that O^+^ ions are distributed in wider range of *L* than H^+^ and He^+^ ions near the plasmapause.

With the recognition of the limitation of the partial densities, it may be of interest to compute the ion composition from values in Fig. 9f and to compare with the results obtained in “Ion composition of oxygen torus” section. Average value of $n'_{{{\text{H}}^{ + } }}$, $n^{\prime}_{{{\text{He}}^{ + } }}$, and $n'_{{{\text{O}}^{ + } }}$ during 02:38–02:46 UT are 2.15 cm^−3^, 0.0989 cm^−3^, and 0.200 cm^−3^, respectively. These values give (H^+^:He^+^:O^+^) = (87.8:4.0:8.2), in which H^+^ concentration is overestimated and O^+^ concentration is underestimated.

In Fig. 9g, it is found that *M*′ is rather constant in the plasmasphere but shows a slight increase during 02:30–02:34 UT as well as a significant enhancement just after 02:34 UT at the plasmapause. We suppose that these increases are real, because lower limits of error bars of *M’* are still higher than the values of *M*′ before 02:34 UT. They may correspond to an inner edge of the oxygen torus. Therefore, we think that the partial density information obtained from the HOPE instrument infers the existence of the oxygen torus.

### Observation by Van Allen Probe A

**Fig. 10:**
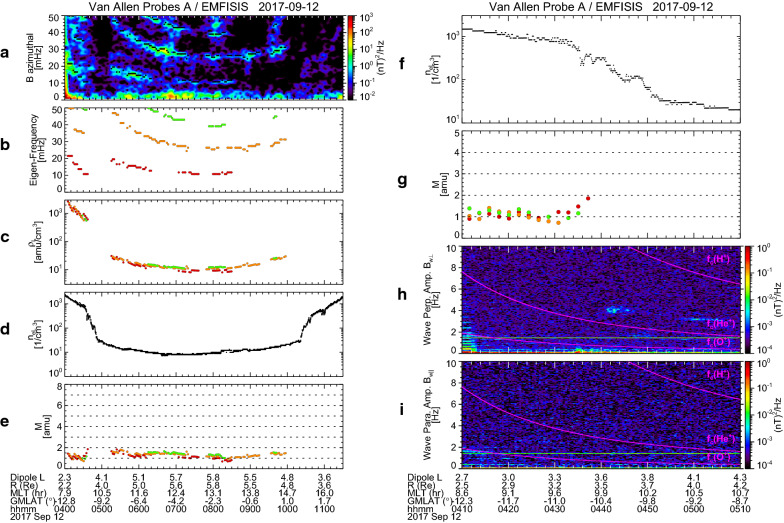
Van Allen Probe A observations of **a** the dynamic power spectrum of the magnetic field variations in the azimuthal component with selected peak frequency indicated by black dots, **b** the selected peak frequency, **c** mass density at the satellite position, **d** electron density at the satellite position, and **e** average plasma mass, during 04:00–11:30 UT on 12 September 2017. Expanded plots during 04:10–05:10 UT of **f** electron density at the satellite position, **g** average plasma mass, and **h**, **i** the dynamic power spectra of wave amplitudes in the directions perpendicular/parallel to the local magnetic field

The Van Allen Probes consist of two identical satellites (Probes A and B) that have almost the same orbits. On 12 September 2017, Probe A was flying behind Probe B by 2 h at a slightly later MLT as shown in Fig. 1a. Thus, it will be of interest to examine data from Probe A during this event. We performed the same analysis for the Probe A data as for the Probe B data. Figure 10a–10e displays the results for 04:00–11:30 UT in the same format as Fig. 4a–e. Profiles of *ρ*_L_, *n*_eL_, and *M* (Fig. 10c–e) do not generally change in comparison with those of Probe B (Fig. 4c–e), although the plasmapause in the afternoon (~ 15 MLT) is more clearly identified at 10:20 UT. This implies that the whole structure of plasma in the inner magnetosphere is rather stable during the 2-h interval between the Probe B and Probe A observations. It should be noted in Fig. 10a that the standing wave signatures are absent at 04:40–05:10 UT, during which Probe A traversed the oxygen torus region that is found by Probe B around 02:40 UT. This may indicate that the standing Alfvén wave in the heavy plasma is damped more quickly. Since we have no information about the standing wave frequencies during this time interval, it is not possible to identify the oxygen torus from the Probe A data; but we expect its existence on the basis of the aforementioned stable structure of the magnetospheric plasma.

Figure 10f–i compiles the expanded plots of *n*_eL_, *M*, and the dynamic power spectra of *B*_w⊥_ and *B*_w||_ from Probe A for 04:10–05:10 UT in the same format as Fig. 6a–d. No data of *M* are given after 04:40 UT, but the oxygen torus is anticipated around 04:40–04:50 UT from the Probe B observations (Fig. 6b). We also calculated *M′* from the Probe A/HOPE data and confirmed the similar slight increase of *M′* during 04:32–04:43 UT, which corresponds to an inner edge of the oxygen torus (not shown here). In Fig. 10h and i, we find wave activity at 4–4.5 Hz only in *B*_w⊥_ during 04:40–04:50 UT. Although the power of this wave is smaller than that of the EMIC wave found in Fig. 6c, its frequency and occurrence region is the same (i.e., ~ 4 Hz, *L* ~ 3.6–3.8, and MLT ~ 9–10 h). Thus, this wave is considered the EMIC wave and displays the simultaneous occurrence with the oxygen torus.

## Conclusions

**Fig. 11:**
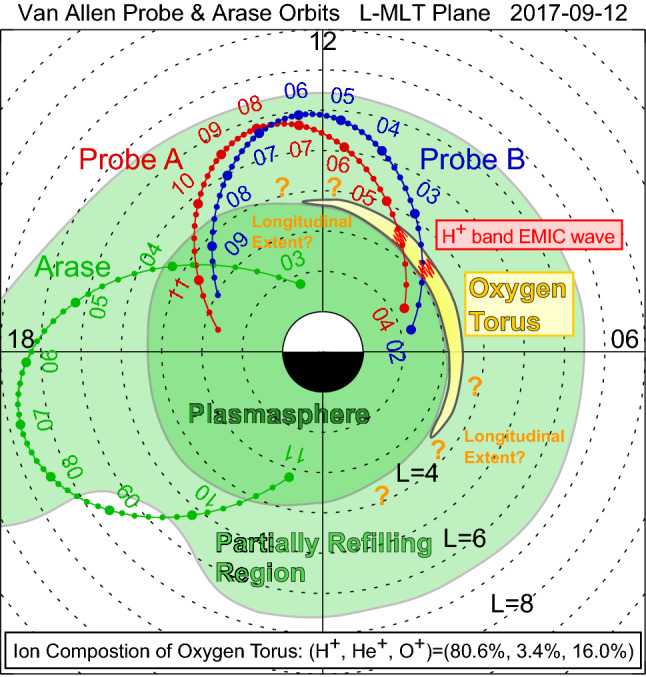
A schematic figure summarizing findings of the present study for the 12 September 2017 event

In this study, we investigate the longitudinal structure of the oxygen torus for a specific event on 12 September 2017, using data from the Van Allen Probe B and Arase satellites. Figure 11 summarizes findings of the present study. Probe B identified the enhancement of *M* up to 3–4 amu at *L* = 3.3–3.6 and MLT = 9.0 h, where is just outside the plasmapause. It flew within the partially refilling region on the dayside and traversed the plasmapause at *L* ~ 3.3 and MLT = 15.7 h. There is no clear enhancement of *M* near the plasmapause on the afternoon side. This is confirmed by the observations from Arase, which traversed the plasmapause at *L* ~ 4.3 and MLT ~ 16.0 h. From these results, we infer that the oxygen torus is not uniform in the longitudinal direction but is skewed toward the dawn, perhaps described more precisely as a crescent-shaped torus or a pinched torus as proposed by Nosé et al. (2015, 2018). In Fig. 11, the crescent-shaped oxygen torus is depicted to have ~ 9 h extent in MLT (yellow area), but its actual longitudinal extent is not yet known from these two-point measurements. Future studies to reveal the longitudinal extent of the oxygen torus may include global imaging of emission from O^+^ ions by an EUV camera carried by satellites (Goldstein et al. 2018) or a numerical simulation of low energy O^+^ ions traveling from the ionosphere to the inner magnetosphere in realistic electric and magnetic field models.

When Probe B identified the oxygen torus in the morning, the H^+^ band EMIC wave was found at approximately 4.5 Hz (red zigzag line). The occurrence of the EMIC wave is coincident with the enhancement of *M*. Probe A also observed a similar EMIC wave in the *L* range where the oxygen torus is expected, although its wave power was smaller than that of Probe B. Using the cutoff frequency of the EMIC wave, we estimate that the oxygen torus for this event was composed of 80.6% H^+^, 3.4% He^+^, and 16.0% O^+^. The linearized dispersion relation for EMIC waves is solved for the 3-component (H^+^, He^+^ and O^+^ ions) plasma. The result shows that both He^+^ and O^+^ ions inhibit EMIC wave growth and the stabilizing effect is stronger for He^+^ than O^+^. Therefore, when $p_{{{\text{H}}^{ + } }}$ or *M* is constant, the plasma with denser O^+^ ions naturally has more tenuous He^+^ ions, resulting in a weaker stabilizing effect (i.e., larger growth rate). On the basis of the Probe B observations, we find that the growth rate becomes larger in the oxygen torus than in the adjacent regions in the plasma trough and the plasmasphere.

## Data Availability

Science data of the ERG (Arase) satellite were obtained from the ERG Science Center operated by ISAS/JAXA and ISEE/Nagoya University (https://ergsc.isee.nagoya-u.ac.jp/index.shtml.en, Miyoshi et al. 2018b). The present study analyzed the OBT L2_v02 data, the MGF L2_v03.03 data, and the PWE-HFA L2_v01.01 data. The Van Allen Probes/EMFISIS data are available at http://emfisis.physics.uiowa.edu. The present study used the EMFISIS L3_v1.6.1 data. Version of the electron number density data at Probe B position is L4_v1.5.19. The Van Allen Probes/HOPE data are available at http://www.rbsp-ect.lanl.gov. The present study used the HOPE L3_v7.4.0 data with the release 4 calibration. The Dst index is provided by the World Data Center for Geomagnetism, Kyoto, and is available at http://wdc.kugi.kyoto-u.ac.jp. The Kp index was provided by J. Matzka at the Helmholtz Centre Potsdam, GFZ German Research Centre for Geosciences and is available at http://www.gfz-potsdam.de/kp-index. The solar wind data are provided from the OMNIWeb (https://omniweb.gsfc.nasa.gov).

## Abbreviations

DE: Dynamic Explorer
EMFISIS: Electric and Magnetic Field Instrument Suite and Integrated Science
EMIC: Electromagnetic ion cyclotron
GMLAT: Geomagnetic latitude
HFA: High-frequency analyzer
HOPE: Helium, Oxygen, Proton, and Electron
IGRF: International Geomagnetic Reference Field
IMF: Interplanetary magnetic field
LMG: Local magnetic
MGF: Magnetic field experiment
MLT: Magnetic local time
OFA: Onboard frequency analyzer
PWE: Plasma wave experiment
RIMS: Retarding ion mass spectrometer
T89: Tsyganenko 1989
UHR: Upper hybrid resonance
